# Supplementary material for: Healthcare waste generation and quantification in public health centres in Addis Ababa, Ethiopia
Source: PLoS One. 2024 Feb 5;19(2):e0295165. doi: 10.1371/journal.pone.0295165 (PMC10843618; doi:10.1371/journal.pone.0295165)
Supplement: Attachment — Submitted filename: review comments.docx [file pone.0295165.s001.docx]

**Healthcare waste generation and quantification in public health centres in Addis**

**Ababa, Ethiopia**

**Menelik Legesse Tadesse**

**Menelik II Medical and Health Science College** Menelik Legesse Tadesse: corresponding author E-mail: menelik3rd@yahoo.com

**ABSTRACT Background**

Healthcare waste management is very important due to its hazardous nature that can cause risk to human health and the environment. Different kinds of therapeutic procedures (surgery, delivery, resection of gangrenous organs, autopsy, biopsy, para- clinical tests, and injections) are carried out in healthcare facilities and result in the production of hazardous substances, including pathological and infectious wastes, sharp objects and chemical materials. Modern methods to dispose of healthcare waste have been introduced to most healthcare institution and yet there is evidence of healthcare waste mismanagement with reference to public health centres in Ethiopia. The aim of the study was study to assess the type of healthcare waste generation and quantification from selected public health centers in Addis Ababa Ethiopia.

**Methods**

An institutional based, cross-sectional quantitative study were conducted in Addis Ababa City	Administration	selected	public	health	centres	to	measure	healthcare	waste generation rate and quantification. Data was collected by using weighing scale to quantify healthcare waste generation rate from 15 health centres. To assure the data quality calibration of weighing scales was made every morning. Data collectors used data sheet to register daily healthcare waste collected data. Microsoft Excel, EPI- INFO TM 7 and IBM SPSS 20 were used for data entry, cleaning and analysis.

**Result**

The mean healthcare waste generation rate was 10.64 ± 5.79 kg/day, of which 37.26% (3.96 ± 2.017kg/day) was general waste and 62.74% (6.68 ± 4.293 kg/day) was hazardous waste from the studied health centres. The mean (±SD) HCW generation rate in each section was 10.63±5.795 kg/day. Increased amounts of HCW (29.93%) were generated from delivery and postnatal case teams while less (0.32%) HCW was generated from Nutrition and growth monitoring case teams. The mean health care waste generation rate in different case teams in the study health centres was statistically significant (χ2 = 229.2, p-value<0.001). The annual mean (±SD) of HCW generation rate per health centre was 3870.53±2109.84kg/year.

**Conclusion**

In this study healthcare waste segregation at source is crucial to improve waste generation and management practices as well to decrease its quantity. The study highlighted key areas that need to be addressed in order to improve service delivery and eliminate or reduce environmental pollution and human health risk.

**Key words**

Healthcare waste generation; Health centres; Case teams; Addis Ababa; Segregation

**BACKGROUND**

Healthcare waste management is very important due to its hazardous nature that can cause risk to human health and the environment. Modern methods to dispose of healthcare waste have been introduced to most healthcare institutions and yet there is evidence of healthcare waste mismanagement with reference to public health centres in Ethiopia.

Healthcare waste (HCW) produced in the course of health care activities entails a higher risk of infection and injuries than municipal waste. Moreover, it poses serious threats to environmental health and requires specific treatment and management prior to its final disposal(1). Different kinds of therapeutic procedures (surgery, delivery, resection of gangrenous organs, autopsy, biopsy, para-clinical tests, and injections) are carried out in healthcare facilities and result in the production of hazardous substances, including

pathological and infectious wastes, sharp objects and chemical materials. These healthcare wastes may carry germs of disease such as hepatitis B and AIDS (2). In developing countries, healthcare waste has not received much attention and has been disposed of together with municipal waste(3). In Ethiopia, improper healthcare waste management is alarming and poses a serious threat to public health(4).

The risk of healthcare waste and its management has become a global cause of concern. Healthcare waste production at hospitals and its management are important issues worldwide(5). Since the mid-1990s the world has experienced a dramatic increase in the amount of hazardous waste generated. At the same time, a vigorous drive for sustainable development and increased environmental awareness are a concern(6).

Healthcare waste has done much damage to the environment and public health and has been the cause of a high death toll from waste-related diseases (7). A severe outbreak of acute respiratory syndrome in Taiwan led to serious steps in managing healthcare waste(5). A comparison of waste classification between China and the European Union, Japan, and the United States of America found that incinerator workers and people living near incinerators had significantly higher levels of dioxins, furans and hydrocarbon compound in their blood and urine(8).

Studies have been conducted on the generation rate and composition of healthcare waste in Africa examined the healthcare waste status in selected healthcare facilities in Lagos State, Nigeria, West Gojam, Ethiopia and Addis Ababa, Ethiopia(4, 9, 10).

Health problems and removing potential risks to human health, health services inevitably create waste that can pose a health hazard in itself (11). The majority of the problems are associated with an exponential growth in the health care sector together with low or non- compliance with guidelines and recommendations(12). For example, in Mauritius, the percentage of healthcare waste has increased significantly since the 1990s due to population growth, increased number and size of healthcare facilities and the use of disposable medical products (13).

There is growing public concern about HCW in Ethiopia, particularly in Addis Ababa(14). The concern is about the lack of appropriate HCW segregation, selection, handling,

storage, transport, treatment and final disposal. A large proportion of HCW consists of solid	and	liquid	waste, which	cause	health	hazards	and	physical	and	natural environmental degradation. This motivated the researcher to conduct this study to assess the quantification of healthcare waste (HCW) in health facilities in Addis Ababa Ethiopia. Between 2011 and 2022, the Addis Ababa City Administration Health Bureau built more than 75 health centres (HC) and two (2) referral hospital. In addition, the expansion of infrastructure and services to hospitals and health centres is also critical for the production of healthcare waste.

**Materials and Methods**

**Study setting and design**

The study setting was Addis Ababa, the capital city of Ethiopia, with the estimated population of 3,194, 999 with a projected annual growth rate of 2.5%(15). Of the population, 1,515.001 (47.41%) were male and 1,679,998 (53%) were female. It is the largest and most populous city in the country. The city has three layers of administration, the city administration at the top, 11 sub-cities administration in the middle and 116

Woredas at the bottom (16). There are 6 referral hospitals and 101 functional public health centres and all are budgeted from the city administration. An institutional based cross- sectional quantitative study was conducted to measure healthcare waste generation rate in order to achieve the objective.

**Source population**

The source population consisted of 95 ~~public health centres~~ from Addis Ababa City

Government Health Bureau.

**Study Population**

The study population for this research was 15 fully functional public health centres selected from 10 sub cities.

**Study units**

All case teams in selected health centers.

**Inclusion and exclusion criteria**

Public health centres which were fully functional during the study period were included. Public health centres which were not functional during the study period not includer in this study.

**Sample Size and sampling techniques**

In Addis Ababa City Administration Health Bureau there are 95 functional health centres in 10 sub cities. To get the exact number of health centres, sampling fraction, i.e. the ratio of the number of units in the sample to the number of units in the reference population (n/N) where n=number of health centres in the sub city and N= total number of functional public health centres in Addis Ababa City Administration Health Bureau, was used to get the number of health centres (Figure 1).

Sample size determined for this study was determined by the following formula n= (Zα/2)2 * δ2/d2 by calculating sample size (N) for Addis Ababa City Administration Health Bureau Public Health Centres.

Standard normal variate of approximately 95% confidence (Zα/2) =1.96;

δ= population Standard Deviation (SD), estimated by sample Standard Error (SE) SE (δ) =SD/√n=0.312

d= margin of error corresponding 95% of certainty; d= (Zα/2)*SE

1.96*0.312 = 0.6115

0.6115 of 9.61= 6.37% (i.e. within 6.37% from 9.61) give the number (see table 1).

**Table 1	Assumptions and given used for sample size calculation for HCW generation study in Addis Ababa City Government health bureau public	health centres, February 2018.**

**d, % of mean**

**D	(Zα/2)	(Zα/2)^2^	SD	SD^2^	d^2^	N**

**# of HCs at 8 days measurement**

**# of HCs at 7 days measurement**

2.0%	0.0481	1.96	3.8416	3.28	10.76	0.0023	17972	2246	2567.43

4.0%	0.3884	1.96	3.8416	3.28	10.76	0.1478	279.67	34.959	39.953

6.37%	0.612	1.96	3.8416	3.28	10.76	0.375	110.228	13.779	15.747

10%	0.961	1.96	3.8416	3.28	10.76	0.924	44.73	5.59	6.39

15%	1.442	1.96	3.8416	3.28	10.76	2.078	19.892	2.487	2.842

**Data collection tool and procedure**

Data was collected by means of measurement, the researcher visited the study health centres to measure the exact amount of HCW types and generated per day in the selected health centres. Data entry sheet was prepared for the daily measurement of the amount of healthcare waste rate and daily patient visit at each health centers for seven (7) consecutive days by trained data collectors. Data were collected for 24 hours from each sampling unit (case teams) in different color-coded polyethylene bags. The bags color was yellow, red and black. Yellow bags contained infectious healthcare waste and yellow safety boxes for sharp wastes. Black bags contained for noninfectious (non- risk) waste. Red bags contained for drugs and radioactive hazardous waste. Each plastic bag was labeled by BANNER brand sticker showed the date and time started, category of waste and room where waste was collected. Total waste per day were measured at each study unit by removing the plastic bags every morning and its weight was measured every day at 09:00 A.M using weighing scale. At least nineteen (19) case teams were chosen; Pharmacy, Laboratory, Focus	Antenatal	Care	(FNAC), Delivery	and	post-natal, Tuberculosis (TB) and Leprosy, Expanded program for Immunization (EPI), Family planning, HIV/AIDS Counselling and Testing (HTC), Antiretroviral Treatment (ART), Medical recording, Nutrition and growth monitoring (NGM), Abortion procedures, Health Management Information System (HMIS), In-patient, Laundry and Adolescence and youth, Emergency Injection and Dressing room and Out Patient Department (OPD).

**Training**

The researcher gave the fieldworkers three days of training in data collection and the data-collection tools as well as the relevant protocols and precautionary measures not to contaminate themselves and others. The researcher verified data and controlled the study.

**Data quality control**

Fifteen data collectors who graduated from a secondary school with Grade 10 certificates were used for measurement of healthcare waste. Eight supervisors who were BSc graduates in Environmental Health or related fields assisted the principal investigator with the data collection. Data collectors and supervisors were trained by the principal investigator. Training manual was prepared on how to address the data collection check list and how to weigh the healthcare waste. The supervisors calibrated the measuring balances and facilitated the collection. Pre-test was done at two health centers of the same structure other than the sampled areas.

A digital weighing scale model Sartorious Basic Type BA 6100 and electronic compact Balance Model EPB-10001 L digital were used to measure healthcare waste at all the selected healthcare facilities. Calibration was done by using a standard of 20g, 100g,

500g and 1000g weighting objects every morning before the actual measurement started. The standard value was recorded for comparison to the daily activities. Daily onsite supervision was made by the supervisors and principal investigator during the actual measurements and tools assuring ethical issue and respondents assuring an animosity.

**Data entry and analysis**

To determine solid healthcare waste generation rate and composition and to select the best fit predictive models to use to precisely estimate healthcare solid waste generation rate, Microsoft Excel, EPI- INFO TM 7 and IBM SPSS 20 were used for data entry, cleaning and analysis. Data analysis was performed separately for each of the health centres which were grouped by category of ownership. Spearman’s correlation; means; Standard Deviation (SD); frequencies; percentages, and graphs were used. To see the effect of the parameters and their confidence levels on the waste generation rate in

healthcare services, analysis of variance (ANOVA) was performed to compare the rate by type of health centre.

**Ethical consideration**

Ethical approval and clearance were obtained from the Higher Degrees Committee, Department of Health Studies, University of South Africa and Addis Ababa City Administration Health Bureau to conduct the study. The researcher has gotten written and verbal consent to begin the study in the respected health centres directors and case teams. Data collectors were provided data collection manual and trained to use Personal Protective Equipment while handling healthcare wastes. The researcher and supervisors were made alert about the provision of medical assistance for sharps and needle prick injuries.

**Results**

**Patient flow in the study health centres**

A total of 13,897 patients visited the selected health centres on the seven (7) consecutive days of data collection. Of these, 1,765 (12.7%) and 1,527 (10.99%) visited Meshoalekia and Filipos health centres, and 474 (3.41%) and 466(3.35%) visited Woreda 9 and Korea Zemachoch health centres, respectively. The mean (±SD) patients per day in all the selected health centres was 132.35±60.621 (Table 2).

**Table 2 Number of patients visited in the study public health centres, Addis Ababa City Government, February 2018**

**Total patients to health centre on 7**

**Name of health centre**

**days	Mean patients to health centre**

Kolfe	1503	214.71

Filipos	1527	218.14

Meshoalekia	1765	252.14

Teklehaymanot	694	99.14

Woreda 1	685	97.86

Kella	620	88.57

**Total patients to health centre on 7**

**Name of health centre**

**days	Mean patients to health centre**

Saris	784	112.00

Korea Zemachoch	466	66.57

Yeka	872	124.57

Goro	1498	214.00

Millennium	689	98.43

Woreda 9	474	67.71

Michew	690	98.57

Sheromeda	901	128.71

Arada	729	104.14

Mean	926.47	132.35

SD	424.35	60.621

**DAILY HCW GENERATION IN HEALTH CENTRES**

The mean (±SD) HCW generation rate was 10.64 ± 5.790 kg/day, of which 3.96 ± 2.017 kg/day (37.26%) was general waste and 6.68 ± 4.293 kg/day (62.74%) was hazardous waste. A high amount of HCW per day was generated at Filipos and Yeka health centres

26.90 kg/day and 16.96 kg/day, respectively. A small amount of HCW generation was recorded at Woreda 9 and Korea Zemachoch health centres 4.71 kg/day and 5.25 kg/day, respectively (Table 3).

All the health centres operated daily and were open for 24 hours and offered services, but Filipos HC had more patients than the others. In this study revealed that Filipos health centre had the highest HCW generation rate with an average of 0.123 kg/patient/day (Table 3).

**Table 3	Daily HCW generation rate in the study public health centres, Addis**

**Ababa City Government, February 2018**

**Healthcare waste, Kg/day**

**Name of health centre**

**Total HCW**

**in 7 days**

**(observed)**

**Mean of HCW Mean (± SD)**

**Mean of general waste (%)**

**Mean of hazardous waste (%)**

Kolfe	67.44	9.63± 15.328

1.45

(15.31%)

8.19

(84.69%)

Filipos	188.30	26.90±31.341

8.00

(29.75%)

18.90

(70.25%)

Meshoalekia	53.88	7.69±11.041

3.79

(49.28%)

3.90

(50.72%)

Teklehaymanot	39.48	5.63±6.077

2.38

(42.14%)

3.26

(57.86%)

Woreda 1	110.11	15.70±19.070

6.60

(41.97%)

9.10

(58.03%)

Kella	66.30	9.47±11.382

4.098

(43.27%)

5.37

(56.73%)

Saris	94.41	13.49±10.446

2.92

(21.66%)

10.57

(78.34%)

Korea Zemachoch	36.57	5.25±7.489

2.44

(46.77%)

2.80

(53.23%)

Yeka	118.71	16.96±17.963

6.86

(40.42%)

10.10

(59.58%)

Goro	68.75	9.82±13.602

2.98

(30.35%)

6.84

(69.65%)

Millennium	40.75	5.82±8.419

2.85

(49.0%)

2.97

(50.99%)

Woreda 9	32.96	4.71±6.303

1.87

(39.81%)

2.83

(60.19%)

Michew	58.35	8.33±7.752	2.41	5.92

(28.97%)	(71.03%)

Sheromeda	65.27258	9.32±15.958

5.29

(56.77%)

4.03

(43.23%)

Arada	76.478	10.93±15.615

5.47

(50.07%)

5.45

(49.93%)

Overall mean	74.51

10.64	3.96

(37.26%)

6.68

(62.74%)

SD	40.532	5.790	2.017	4.293

**Types and amount of HCW generation**

The results for HCW collected weekly from the study health centres varied in amount of HCW generation. The average value for HCW in each health centre and standard deviation as error bar. The findings indicate significant variations in the HCW generation rates (Figure 2).

The types of hazardous waste generated by the study health centres were sharps, infectious, pathological (placenta and blood) and pharmaceutical. The mean (±SD) generation rate of sharps, infectious, pathological and pharmaceutical waste in each health centre was 0.97± 1.031 (14.63%), 3.23± 2.603 (48.72%), 2.17± 1.917 (32.73%) and 0.26± 0.342 (3.58%) kg/day, respectively (Table 4).

In most of the health centres, the generation of hazardous HCW was high. For example, Filipos HC generated 18.89 kg/day; Saris HC generated 10.57 kg/day, and Yeka HC generated 10.11 kg/day. The lowest amounts of hazardous HCW generated were from Korea Zemachoch HC (2.81 kg/day), Woreda 9 HC (2.83 kg/day) and Millennium HC (2.97 kg/day) (Table 4).

Infectious and pathological waste comprised 81.3% of the hazardous waste; Sharps, Infectious, Pathological and pharmaceutical wastes were 15%, 49%, 33% and 4% respectively (Figure 4).

**Table 4. Distribution of type and amount of daily hazardous HCW generation rate**

**in the study public health centres, Addis Ababa City Government, February 2018**

**Name of health centre**

**Sharps**

**Kg/day**

**Infectious**

**Kg/day**

**Pathological**

**Kg/day**

**Pharmaceutical**

**Kg/day**

**Total hazardous waste Kg/day**

Kolfe	0.73	0.19	5.77	0.37	7.06

Filipos	4.03	11.59	3.28	0	18.89

Meshoalekia	0.71	2.55	0.65	0	3.90

Teklehaymanot	0.78	1.11	1.37	0	3.26

Woreda 1	0.51	4.07	4.30	0.21	9.13

Kella	0.399	2.67	2.09	0.21	5.37

Saris	2.51	4.71	2.83	0.51	10.57

Korea

Zemachoch

0.64	1.91	0.26	0	2.81

Yeka	1.06	4.03	4.37	0.64	10.11

Goro	0.28	1.88	4.68	0	6.84

Millennium	0.68	1.96	0.33	0	2.97

Woreda 9	0.36	1.94	0.54	0	2.83

Michew	0.69	3.24	1.78	0.43	6.14

Sheromeda	0.396	3.14	0.26	0.23	4.03

Arada	0.82	3.42	0	1.21	5.46

Average	0.97	3.23	2.17	0.25	6.63

SD	1.031	2.603	1.917	0.34	4.274

**Daily HCW generation rate in different case teams**

In different case teams, the HCW generation rate varied. The mean (±SD) HCW

generation rate in each section was 10.63±5.795 kg/day. Increased amounts of HCW

(29.93%) were generated from delivery and postnatal case teams while less (0.32%) HCW was generated from NGM case teams (Table 5).

The mean healthcare waste generation rate in different case teams in the study health centres was statistically significant (χ2 = 229.2, p-value<0.001) (Table 5).

**Table 5 Distribution and daily HCW generation rates by point of source in the study public health centres, Addis Ababa City Government, February 2018**

**Case teams	Healthcare waste (Kg/day) Mean± (SD)**

**Percent	Mean rank***

OPD (Outpatient department)

0.59±0.390	5.63	194.8

Pharmacy	0.99±0.636	9.28	225.43

Laboratory	1.76±1.094	16.59	255.53

Emergency and triage	1.085±0.893	10.21	222.60

Injection and dressing	0.64±0.537	5.99	181.1

FNAC (Focus antenatal care)

0.25±0.227	2.39	141.7

Delivery and post-natal	3.18±2.557	29.93	247.8

TB and Leprosy	0.18±0.173	1.66	119.73

EPI (Expanded program for Immunization)

0.54±0.607	5.07	180.23

Family planning	0.32±0.300	3.05	156.07

HTC (HIV Testing and counselling)

0.18±0.125	1.70	123.87

ART (Anti-Retroviral

Treatment)

0.34±0.334	3.22	142.63

Medical recording	0.21±0.145	1.97	134.33

NGM (Nutrition and growth monitoring)

0.03±0.068	0.32	54.03

Abortion procedures	0.038±0.086	0.36	53.17

HMIS (Health Management and Information System)

0.041±0.081	0.39	56.50

In-patient	0.077±0.119	0.72	73.73

Laundry	0.14±0.111	1.34	109.43

Adolescence and youth	0.019±0.057	0.18	44.6

Mean	10.63

SD	5.795

*X^2^= 229.196, p<0.001, df=18

**Estimated annual HCW generation rate**

The estimation of HCW generation rate per year can be calculated in two ways. Firstly, by the annual patient flow and mean HCW generation rate per patient per day (the assumption was each patient who visited the health centre might generate the same amount of HCW throughout the year).

**Total HCW Generation per year =** Mean HCW generation in kg per patient per day* Number of patients flow in a year

Secondly, by using the HCW generation rate per day (kg/day) and number of days in the year (the assumption was the mean of HCW per day might represent throughout 365 days).

The mean (±SD) patient flow per day per health centre was 132.35±60.621. The mean

(±SD) HCW generation rate per health centre was per day or 10.63 ± 5.796 kg/day (Table

6).

The	annual	mean	(±SD)	of	HCW	generation	rate	per	health	centre	was

3870.53±2109.84kg/year using the first method and 3881.14±2195.01 kg/year using the second assumption. There was a slight variation of the annual HCW generation rate in both assumptions (Table 6).

**Table 6 Estimated total HCW generation rate per year in the study public health**

**centres, Addis Ababa City Government, February 2018**

**Name of health centre**

**Patient flow in 2018**

**Patient flow per day 2018**

**Mean HCW**

**kg/day**

**Mean HCW**

**g/pat/day**

***Total HCW kg/year**

****Total HCW kg/ year**

Kolfe	78371	214.71	9.63	44.85	3514.95	3514.94

Filipos	79622	218.14	26.90	123.31	9818.5	9818.19

Meshoalekia	92032	252.14	7.7	30.53	2810.5	2809.74

Teklehaymanot	36187	99.14	5.64	56.89	2058.6	2058.68

Woreda 1	35718	97.86	15.73	160.74	5741.45	5741.31

Kella	32329	88.57	9.47	106.93	3456.55	3456.94

Saris	40880	112	13.49	120.42	4923.85	4922.77

Korea Zemachoch	24299	66.57	5.22	78.48	1905.3	1906.99

Yeka	45469	124.57	16.96	136.13	6190.4	6189.70

Goro	78110	214	9.82	45.90	3584.3	3585.25

Millennium	35926	98.43	5.82	59.14	2124.3	2124.66

Woreda 9	24716	67.71	4.71	69.54	1719.15	1718.75

Michew	35979	98.57	8.34	84.56	3044.1	3042.38

Sheromeda	46981	128.71	9.32	72.44	3401.8	3403.30

Arada	38012	104.14	10.93	104.91	3989.45	3987.84

Average	48308.73	132.35	10.65	86.32	3885.55	3885.43

SD	22126.89	60.621	5.794	37.731	2114.74	2114.50

*Total HCW generation rate in kg per year=HCW generation rate in kg per day * 365

**Total HCW generation rate in kg per year= (HCW generation rate in grams per patient per day * Number of annual patients flow)/1000

**Patient flow and HCW generation comparison**

The patient flow and HCW generation rate and types, such as general and hazardous waste (sharps, infectious, pathological and pharmaceutical waste), among the study health centres were compared using the Kruskal-Wallis test to check for the presence of

significant differences among their values. There was no statistically significant difference for the mean patient flow (x^2^=14.504, p-value=0.106) and the mean general waste (x^2^=22.631, p-value=0.067), but there was a statistically significant difference for mean of healthcare waste (x^2^=9.421, p-value=0.803) and the mean hazardous waste (x^2^=35.819, p-value=0.001) among the study health centres (see Table 7).

**Table 7 Comparison of patient flow and HCW generation rate and types among the study public health centres, Addis Ababa City Government, February 2018**

**Name of health centre**

**Mean rank**

**Patient flow	Total HCW	Non-hazardous**

**HCW**

**Hazardous HCW**

Kolfe	214.7	9.63	1.45	8.19

Filipos	218.1	26.90	8.00	18.90

Meshoalekia	252.1	7.70	3.79	3.90

Teklehaymanot	99.1	5.63	2.38	3.26

Woreda 1	97.9	15.70	6.60	9.10

Kella	88.6	9.47	4.10	5.37

Saris	112	13.49	2.92	10.57

Korea Zemachoch	66.6	5.25	2.44	2.80

Yeka	124.6	16.96	6.86	10.10

Goro	214	9.82	2.98	6.84

Millennium	98.4	5.82	2.85	2.97

Woreda 9	67.7	4.71	1.87	2.83

Michew	98.6	8.34	2.41	5.92

Sheromeda	128.7	9.32	5.29	4.03

Arada	104.1	10.93	5.47	5.45

Chi-square	14	22.631	35.819	9.421

Asymp. Sig. 0.450	0.067	0.001	0.803

Degree of freedom was 14. The extent or strength of linear relationships between the number of patients and amount of HCW generation rate was checked using the Spearman’s rank correlation coefficient (rs) in all the health centres. The Spearman’s rank correlation coefficient showed a positive linear relationship: as the number of patients increased, HCW also increased in all the study health centres. A strong linear relationship was observed at Filipos and Meshoalekia health centres: the Spearman’s correlation coefficient was 0.964 and 0.964, respectively which is far from a perfect linear relationship at Spearman’s correlation coefficient value (rs=1). A strong linear relationship was not observed at Saris, Kolfe,Teklehaymanot and Korea Zemachoch health centres: 0.126,

0.321, 0.342, and 0.342, respectively, which is far from a perfect linear relationship at

Spearman’s correlation coefficient value (rs=1) ( Table 8).

**Table 8 Correlation of patient numbers and quantity of waste generated in the study health centres, Addis Ababa City Administration, February 2018**

**Name of health centre	Spearman’s rank correlation coefficient (rs)**

Kolfe	0.321

Filipos	0.964

Meshoalekia	0.964

Teklehaymanot	0.342

Woreda 1	0.559

Kella	0.631

Saris	0.126

Korea Zemachoch	0.342

Yeka	0.643

Goro	0.607

Millennium	0.893

Woreda 9	0.357

Michew	0.571

Sheromeda	0.607

Arada	0.429

**Score of Hazardous and non-hazardous waste HCW**

Hazardous HCW and non-hazardous HCW had different lower scores and hazardous HCW was higher. The median for hazardous HCW was higher than for non-hazardous waste. The first quartile (Q1) was equal to 47.32 kg/7days to the total HCW, about 25% of the total HCW was lower than 47.32 kg/7 days and about 75% was above 47.32 kg/7 days. Regarding nonhazardous HCW, the first quartile (Q1) was equal to 17.0 kg/7 days about 25% of nonhazardous HCW was lower than 17.0 kg/7 days. The total HCW showed a lower cut-off -9.87 and an upper cut-off 75.57 kg/7 days. The hazardous HCW also had a lower cut-off -28.1 kg/7 days and an upper cut-off 32.41 kg/7 days (Figure 5).

**Relationship between HCW generated in kg/day and the daily number of patients**

Relationship between HCW daily amounts generated in kg/day and the daily number of patients visited was compared by a scatter plot in study health centres. A linear trend was evident between the amount of total HCW generation and total number of patients (statistically significant, P<0.067; R2=0.135). Therefore, the number of patients that visited the health centres daily could be used as a predictor of HCW generation rates in the health centres. This R2 also showed a moderate linear relationship between the number of patients that visited the health centres and the amount of HCW generated. In particular, 13.5% of the variability among the observed values of HCW generation in the

7 days of HCW measurement was explained by the linear relationship between the total number of patients that visited the health centres and generation of HCW (Figure 6).

**Discussion**

In this study the mean HCW generation rate was 10.64 + 5.79 kg/day, of which 37.26% (3.96 + 2.017kg/day) was general waste and 62.74% (6.68 + 4.293 kg/day) was hazardous waste other similar study done in Bench Maji Zone Ethiopia, health centres showed out of the total HCW generated, more than half (57.9%) was general or non-risk HCW, and the remaining 42.1% was hazardous healthcare waste(17). In both studies the amount of HCW generated in the studied health centers was different from WHO’s norm

WHO report in health facility setting, general was 85% while hazardous was 15%(18). which may be attributed to different factors such as poor waste segregation practice, difference in services provided, patient flow, economy, available waste management system and seasonal factors(17).

The mean health care waste generation rate in different case teams in the study health centres was statistically significant (χ2 = 229.2, p-value<0.001). This may be due to the similarity of the kinds of health service had been given among study health centers and resource	allocation. Meanwhile	there	was	no	statistically	significance	difference (X2=14.504, p-value=0.106) in patient flow between study health centers. Similar study done in Bench Maji Zone Ethiopia also showed there was a significant difference in the total healthcare waste generation rate between studied health centers with (χ2 = 20.068, p value= 0.017). This may be attributed to the status of patient flow and resource allocation to the health centres. However, there was no significance difference (χ2=4.586, p-value=0.869) for patient flow between studied health centers(17).

The study done in Adama health centers the daily average outpatient flow of all health centers was 171 patients per day(19). In this study the daily average health centres patient flow was 132 this might be of the proportion of health centres per population and the season during the study done.

The amount of healthcare waste generation rate in different case teams in this study varied. High quantity 29.93% of HCW was found in delivery and postnatal case team whereas fewer amounts 0.18% of healthcare waste was generated at adolescence and youth case team. Similar study done in Adama Zone Ethiopia showed the highest quantity of HCW in all studied health centers was attributed to delivery rooms, which accounted for 39.8% of HCW, and the least amount was recorded at the voluntary counseling and testing department, which accounted for 7.3% of HCW(19).

The annual mean (±SD) healthcare waste generation rate estimate per health center in this study was 3870.53 ± 2109.84 kg/year. The estimation of annual mean healthcare waste generation rate can vary in the assumptions. Similar study done in Addis Ababa health centres was 3501.86 ± 1204.29 kg/year(4). This may be due to the variation of annual patient flow and seasonal variation. However, the assumption was the preferable

method to estimate annual health care waste generation rate because the mean of annual healthcare waste was determined by annual patient flow within the health center.

The mean healthcare waste generation rate in gram per patient per day per health center in this study was 86.32. It was higher than the study done in Addis Ababa, Ethiopia, health centers 57.37g/per day per patient (4). It was also different from another study done in Sylhet city, Bangladesh in diagnosis center and higher clinics, the mean healthcare waste generation rate was 0.041 kg/patient/day(20). This variation may be due to geographical location, season of the year, availability of different facilities, social status of the patients (i.e., income, living standard, awareness about disease), healthcare waste management and legislation of system of the country.

**Conclusion**

The mean HCW generation rate was 10.64 + 5.79 kg/day, of which 37.26% (3.96 +

2.017kg/day) was general waste and 62.74% (6.68 + 4.293 kg/day) was hazardous waste from the surveyed health centres. The mean of healthcare waste generated in all health centers was greater in amount when compared from similar study in the country.

During the HCW collection survey week, the study found that more HCW was generated from health centres with no public hospital nearby. The smallest amounts of HCW generated were from health centres that used nearby public hospitals (Table 4).

The study findings contribute to all health facilities to prepare HCW management plan in order to minimize generation, promote proper segregation, collection, transportation and 0final	disposal. Training	program	at	all	levels	such	as	individuals, interpersonal, organizational and community level need to be standardized and the effectiveness thereof assessed regularly by the Federal Ministry of Health Ethiopia and Addis Ababa City Government Health Bureau.

To improve operations healthcare facilities should appoint infection control teams / committees that include specialists to occupational and environmental health and waste management experts. The findings of the study should contribute to the achievement of the United Nations (21) sustainable development goals (SDGs) for 2016-2030, which are aimed at bringing about a sustainable world and protecting the planet such as SDG 3: Good health and well-being, SDG 6: Clean water and sanitation, SDG 8: Decent work

and economic growth, SDG 12: Responsible consumption and production and SDG13: Climate action.

**Limitation of the study**

This study has the following limitation: the study conducted was cross-sectional and couldn’t identify causality. The study was conducted in public health centres healthcare waste generation and couldn’t represent healthcare waste outside the public health centers (private HCF). The study is conducted the generation rate on healthcare waste other studies should also address HCW issues is very important.

**Acknowledgment**

First, I would like to express my deepest gratitude to Professor Bethabile Lovely Dolamo for her unreserved support throughout the study period. I sincerely thank University of South Africa, Menelik II Medical and Health Science College, Addis Ababa City Government Health Bureau, heads of the study health centers for their unreserved cooperation during data collection time. My deepest gratitude also goes to all data collectors and supervisor for their commitment during data collection. I would like to thank my beloved wife Alemnesh Mude, daughters Bezawit Menelik and Hermela Menelik for their patience during the study period.

**Competing Interest**

The author declares that there is no financial or non-financial conflict of interest related to this article.

**Authors’ contribution**

Tadesse ML Drafted the proposal, organized the research team and data collection, performed statistical analysis and drafted the manuscript.

**Authors’ information**

Menelik II Medical and Health Science College, Addis Ababa, Ethiopia.

*Corresponding author: menelik3rd@yahoo.com

**References**

1. Shahryar Sorooshian SAMA, Amin Teyfouri Technology and management Kuala Lumpur: Lulu

Press; 2014. 258 p.

2. Prüss-Ustün A, Rapiti, E & Hutin, Y. . Estimating the global burden of disease attributable to contaminated sharp injuries among health care workers. American Journal of Industrial Medicine.

2005;48(6):482-90.

3. Pichtel J. Waste management practices: municipal, hazardous, and industrial. Second Edition ed. Boca Raton, FL: CRC Press; 2014. 682 p.

4. Tadesse MK, A. . Healthcare waste generation and management practice in government health centres of Addis Ababa, Ethiopia. BMC Public Health. 2014;14(1221).

5. Cheng Y, Li, K & Sung, F. Medical waste generation in selected clinical facilities in Taiwan. Waste

Management. 2010;30(8):1690-5.

6. Ketlogetswe C, Oladirang, MT & Foster, J. . Improved combustion process in medical waste incinerators for rural application. African Journal of Science and Technology. 2004;5(2):67-72.

7. Kumar A. Human development and natural resources. In: Forsythe D, editor. Encyclopaedia of

Human Rights. London: Oxford University Press; 2006.

8. Wen X, Luo, Q, Hu, H, Wang, N, Chen, Y, Jin, J, Hao, Y, Xu, G, Li, F & Fang, W. . Comparison research on waste classification between China and the EU, Japan, and the USA. Journal of Material Cycles and Waste Management. 2014;16(2):321-34.

9. Longe E. Healthcare waste management status in Lagos State, Nigeria: a case study from selected health care facilities in Ikorodu and Lagos metropolis. Waste Management and Research.

2012;30(6):562-71.

10. Azage MK, A. . Healthcare waste generation and its management system: the case of health centres in West Gojjam zone, Amhara Region, Ethiopia. Ethiopian Journal of Health Development.

2010;24(2):119-26.

11. Chartier Y, Emmanuel, J, Pieper, U, Prüss, A, Rushbrook, P, Stringer, R, Townend, W, Wilburn, S

& Zghondi, R. . Safe management of wastes from health-care activities. 2nd edition ed. Geneva: WHO;

2014.

12. Acton Q. Issues in global environment: pollution and waste management. Atlanta, GA: Scholarly

Editions; 2011.

13. Mohee R. Medical wastes characterization in healthcare institutions in Mauritius. Waste

Management. 2005;25(6):575-81.

14. Association EPHL. Laboratory safety practice in Ethiopia. Addis Ababa: Ethiopian Public Health

Laboratory Association (EPHLA), 2014.

15. Agency CS. Population size by sex, area and density by region, zone and Wereda. Addis Ababa, Ethiopia: Central Statistic Agency, 2012.

16. Bureau AACACA. Addis Ababa city at a look. Addis Ababa, Ethiopia: Government Printer; 2016.

17. Asrat Meleko TT, & Andualem Henok Assessment of Healthcare Waste Generation Rate and Its

Management System in Health Centers of Bench Maji Zone. Ethiop J Sci. 2018;28(2):125.

18. Organization WH. Waste from Healthcare Activities. Geneva Switzerland: WHO; 2000.

19. Cherinete SFHW. Healthcare Waste Generation and Management in Public Healthcare Facilities

in Adama, Ethiopia. Journal of Health and Pollution 2016;6(10):64-73.

20. Prüss A, Giroult, E & Rushbrook, P. Safety management of wastes from health-care activities. Geneva: WHO; 1999.

21. program Und. Sustainable Development Goals. New York, United States of America: UNDP;

2015.

Figure	Click here to access/download;Figure;Figures 2 For PLOS ONE.docx

**FIGURES**

**Figure 1 Number of health centres selected from ten sub-cities of Addis Ababa**

**City Government, February 2018**

250

200

150

**Average HCW (Kg/day)**

100

50

0

**Tyes of HCW**

Non hazardouswaste	Hazardous Waste	Total Hazardous waste

**Figure 2 Error bar of average HCW generation rates in the study public health centres, Addis Ababa City Government, February 2018**

**32.51, 33%**

**3.81, 4%**

**, 14%**

**14.595**

Sharp Infectious Pathological

Pharmaceutical

**48.41, 49%**

**Figure 3 Composition, contribution and generation of hazardous HCW in the study health centres, Addis Ababa City Administration, February 2018**

**Figure 4 Hazardous HCW generation rate (kg/day) in the study public health centres, Addis Ababa City Government, February 2018**

_30_	Total HCW	Hazardous HCW Nonhazardous HCW	Linear (Total HCW)

Linear (Hazardous HCW)	Linear (Nonhazardous HCW)

25

20

y = 0.0351x + 6.0016

15	R² = 0.1348

y = 0.0298x + 2.7333

10	R² = 0.1773

Mean HCW Kg/day

5	y = 0.0053x + 3.2631

R² = 0.0251

0

0	50	100	150	200	250	300

Daily Patients/Visitors

**Figure 5 Relationship between HCW generated in kg/day and the daily number of patients at the study public health centres, Addis Ababa City Government, February 2018**
