# Supplementary material for: Healthcare waste generation and quantification in public health centres in Addis Ababa, Ethiopia
Source: PLoS One. 2024 Feb 5;19(2):e0295165. doi: 10.1371/journal.pone.0295165 (PMC10843618; doi:10.1371/journal.pone.0295165)
Supplement: Attachment — Submitted filename: Response to reviewers May 2023.docx [file pone.0295165.s002.docx]

**Response to reviewers**

Dear Academic editors and reviewers thank you for your constructive comments.

**Comments to the author part**

Comment #1 what is the importance of discussing the methods of health care waste disposal system although your title is on generation and quantification of HCW?

Did you assess the disposal system of HCW in this study?

**Dear reviewer generation and quantification is part of the system, but I didn’t assess in this study.**

Comment #2: Rewrite this sentence again there is a repetition of the words "The aim of the study was study to assess the type of healthcare waste generation and quantification from selected public health centers in Addis Ababa Ethiopia."

**Dear reviewer I corrected the repetition.**

Comment #3: You assessed the type of healthcare waste generation. What type is it? Did you study the rate of HCW generation or the type of HCW generation?

**Dear reviewer I focused on solid healthcare waste.**

Comment #4: Is the SPSS appropriate for data entry?

**Dear reviewer no, Microsoft Excel 2016 and EPI- INFO TM 7 were used for data entry and IBM SPSS 20 for analysis.**

Comment #5: Based on research findings 3/4 of HCW is general waste. But you come up with 62.74% hazardous waste. What is the reason behind this finding? Can you give a justification?

**Dear reviewer the reason behind is there is no segregation at the point of generation.**

Comment #6: Your objective is about waste generation rate and quantification, but your conclusion was for waste segregation and its management practice.

**Dear reviewer I have corrected the sentence.**

Comment #7: How can waste segregation improve the generation rate? Which one comes first?

The conclusion part of both the abstract and the revised manuscript is corrected according to the comment gave by the reviewer.

**Dear reviewer I have corrected according to the comment.**

Background:

Comment #1: For paragraph one where is its source? It is better to cite particularly the source.

All paragraphs did not explain the background information regarding the waste generation rate and its quantification. But it explains the practice of HCW management, its risks, and consequences for the environment and human beings. I suggest you rewrite the background and to full fill in some incomplete and nonsense paragraphs like paragraph five.

**Dear reviewer I have changed background start paragraph and citation also indicated in the paragraph.**

Materials and Methods:

Comment # 1: Why for you focused to study on a Health center setting only? Why not other hospitals?

**Dear reviewer my study focused on public health centres I will do hospital healthcare waste generation later in other study.**

Comment # 2: What by mean functional health centers? Were there any health centers nonfunctional at the study time?

**Dear reviewer there were not functional health centres during the study period because of infrastructural delay.**

Comment # 3: Your study's Inclusion and exclusion criteria are the same. Try to identify them and justify what you have done in your study.

**Dear reviewer during the study the inclusion criteria was public health centres which were fully functional. Public health centres which not functional during the study period were not included in this study.**

Comment # 4: What was your role in data collection? Is it ethical for the researcher to measure the study variables?

**Dear reviewer my role in data collection was to control the over all aspects of the study by following the supervisors and data collectors.**

Comment # 5: How you assured the health and safety of data collectors?

**Dear reviewer all supervisors and data collectors were trained the health and safety and reporting of any accident during the study period.**

Comment # 6: How you categorized wastes as hazardous and general?

**Dear reviewer the category of hazardous waste was include sharps, infectious, pathological and pharmaceutical wastes. The non hazardous wastes include general waste (non-contaminated like papers, plastics, cloths, left over food and any dry waste).**

Results and Discussion:

As I have described in the part of Abstract how can segregation affect the generation rate?

Rewrite this limitation part sentence again [The study was conducted in public health centres healthcare waste generation and couldn’t represent healthcare waste outside the public health centers (private HCF)].

Conclusion:

Is it possible to conclude about the collection, segregation, and transportation and disposal system of HCW with your study objective, design, and findings?

**Dear reviewer I corrected the result and discussion part, but I didn’t change table 1 it is important for the readers how to calculate the sample size for the study health centres also I didn’t change table 2 because it help the readers about how many patient visitors reached in the health centres each day during the study period.**

**Dear reviewer I corrected the legends for the figures and wrote separately and also corrected the figures 3 and 6 to texts.**

To note that the author followed the manuscript writing according to submission guideline PLOS ONE.

It is my pleasure to see your response soon.

**Tadesse, Menelik Legesse**
