# Supplementary material for: Healthcare waste generation and quantification in public health centres in Addis Ababa, Ethiopia
Source: PLoS One. 2024 Feb 5;19(2):e0295165. doi: 10.1371/journal.pone.0295165 (PMC10843618; doi:10.1371/journal.pone.0295165)
Supplement: Attachment — Submitted filename: Response to reviewers September 2023.docx [file pone.0295165.s003.docx]

**Response to Academic editor and reviewer**

Dear Academic editors and reviewers thank you for your constructive comments.

**Reviewer #3 comment**

**Abstract**

Most points raised by the reviewers; Abstract, Background, Methods, Results and Conclusion part are amended to the revised manuscript.

**Background**

The background paragraphs break to 5 as per the comment. It is amended and include citation within 2020 and 2023. The author tried to indicate gaps link to the study objective to the revised manuscript.

**Material methods**

In the statistical analysis section the detailed of software aspects amended as per the comment provided to the revised manuscript.

**Discussion**

As the reviewer comment the author amended theoretical, empirical, policy and method concern. The recent citations also included as per the reviewer comment to the revised manuscript.

**Conclusion**

The conclusion part of both the abstract and the revised manuscript is corrected according to the comment gave by the reviewer**.**

**References**

The reference part also corrected as the format of the journal in the revised manuscript

**Others**

The legend, caption, and foot notes amended as per the comments in the revised manuscript.

**Reviewer #4**

**Background**

The author include the research gap and objective in the background section to the revised manuscript.

**Result section**

As the comment by the reviewer the sub-headings are corrected to the revised manuscript. Inclusion and exclusion criteria included as per the journal format.

The study design, study units and population amended as per the comment by the reviewer in the revised manuscript. The sample size and sampling technique also corrected with citation in the revised manuscript.

The discussion and conclusion amended and provided recent citations and justification in the revised manuscript.

Table 1 presented as it is merged and the narrative of table also amended based on the data of the patient and the healthcare waste amounts in the revised manuscript.

Table 6 Caption corrected as per the comment by the reviewer to the revised manuscript.

Number of tables reduce to six in the revised manuscript.

The language for the narrative also amended to the revised manuscript.

To note that the author followed the manuscript submission guideline for PLOS ONE.

It is my pleasure to see your response soon.

Tadesse, Menelik Legesse
